# Botanicals and Phosphonate Show Potential to Replace Copper for Control of Potato Late Blight

**DOI:** 10.3390/jof3040065

**Published:** 2017-11-24

**Authors:** Hans-Rudolf Forrer, Susanne Vogelgsang, Tomke Musa

**Affiliations:** Agroscope, Reckenholzstrasse 191, 8046 Zurich, Switzerland; susanne.vogelgsang@agroscope.admin.ch

**Keywords:** *Phytophthora infestans*, induced resistance, phosphonic acid, medicinal plant, phosphite, organic, residue

## Abstract

Potato late blight (PLB) caused by *Phytophthora infestans* (Pi) is the most harmful disease in potato production worldwide. In organic farming, copper is used despite its persistence in soil and toxicity to soil organisms. To replace copper, suspensions of powders from three promising botanicals, including bark of buckthorn (*Frangula alnus*, FA), roots of medicinal rhubarb (*Rheum palmatum*) and galls of the nutgall tree (*Galla chinensis*), were tested in multi-year field experiments. The current study shows for the first time that botanicals could replace copper under field conditions and best PLB reduction on leaves was achieved with FA, reaching a level close to that of 2 to 3 kg copper per hectare and year. Better results than with copper were achieved with Phosfik^®^ (Ph), a phosphonate-based product. For both FA and Ph, the mode of action is based on induced resistance, for Ph also on direct fungicidal effects. A disadvantage of Ph is the accumulation of residues in potato tubers. Nevertheless, two to three applications with 2 to 3 L/ha of Ph would be feasible to not exceed a minimal risk level (MLR) of 20 mg/kg of phosphorous acid as proposed by the European Food Safety Authority. Due to an excellent environmental profile and a complex mode of action counteracting Pi resistance, phosphonate-based products would be most suitable for sustainable PLB management in integrated pest management (IPM) programmes.

## 1. Introduction

Potato late blight (PLB) caused by *Phytophthora infestans* (Pi) is the most harmful disease in potato production worldwide and, in the European Union (EU), causes estimated annual losses of more than €1,000,000,000 [1]. Based on the biology of Pi with asexual and sexual reproduction, an extremely fast reproductive growth rate and the change to more aggressive Pi populations renders the control of PLB with fungicides or the breeding of less susceptible potato varieties most challenging [2]. In Switzerland, control of PLB in conventional potato production relies primarily on the use of synthetic fungicides with about seven to 10 applications per season. However, due to the fast development of resistance problems, as has been observed with systemic phenylamide fungicides in 1980, the agro-chemical industry and its fungicide resistance action committee (FRAC) made strong efforts to reduce the risk of pathogen resistance, including the combination of PLB-fungicides with different modes of action, and by restricting the number of applications for particular fungicides [3].

In organic agriculture, the main approach is the implementation of cropping factors to prevent plant diseases, as opposed to direct control measures. The preventative methods include optimised crop rotation, the use of disease-free seed tubers and the cultivation of potato varieties with low PLB susceptibility. However, despite the availability of such potato varieties, they were as of yet rarely introduced into practice since they did not fulfil market requirements [4].

The only synthetic direct control measure allowed in organic potato production is the use of copper-based products. In their review on the impact of agricultural inputs, Buenemann et al. [5] stated that copper fungicides can have drastic negative effects on soil organisms. Based on such reports about the toxicity of copper, the EU proposed a ban of copper fungicides as early as 2002. Since such a ban would have threatened the feasibility of organic potato production, it was not imposed as of now, but this initiative led to intensified research for new approaches to reduce the risk of PLB attacks and for natural products to replace or reduce the use of copper [6]. Subsequently, substantial investigations were conducted to develop and evaluate the efficacy of copper-free products (CFP) for organic potato production. In extensive in vitro evaluations and in experiments with Pi-inoculated detached potato leaves, using plant-based products, compost, fungal and bacterial extracts, none of the treatments was as effective as copper [7,8]. In a Swiss study with 53 commercial or experimental CFPs, 27 of the best performing CFPs were tested in field experiments, however, no significant effects on PLB control or on yield increase were observed [9]. Other research focused on preparations based on medicinal plants. In growth chamber experiments with potted potato plants, promising efficacies were observed with ethanol extracts of *Galla chinensis* (GC), roots from *Rheum palmatum* (RP) and bark from *Frangula alnus* (FA). Nevertheless, in contrast to ethanol extracts, significant and far higher reductions of foliar blight were achieved when aqueous suspensions of finely ground plant material were applied [10].

The mode of action and the efficacy of GC, RP and FA were also evaluated in experiments with grapevine downy mildew caused by the oomycete *Plasmopara viticola* [11]: Out of 21 potential elicitors of grapevine defence mechanisms, RP and FA performed best and inhibited the pathogen by the stimulation of callose and the synthesis of the antifungal stilbene δ-viniferin. In addition, extracts of GC showed important antifungal effects without induction of plant defence mechanisms.

In organic viticulture, the challenge to control *P. viticola* is quite similar to the one with Pi in potatoes as direct control is largely based on copper fungicides and the existing lack of effective alternatives [12]. Another research group investigated 112 different CFPs under greenhouse and partially under field conditions. However, and similar to the situation in PLB control, the authors stated that alternative products were not effective enough to replace copper [13].

Nevertheless, promising results were observed with the application of phosphonic acid (H_3_PO_3_): In grapevine field trials in 1986 in Australia, efficacies in the control of *P. viticola* were equivalent to those of the phenylamide fungicide Ridomil^®^ (Syngenta International AG, Basel, Switzerland) [14]. Furthermore, in potato field experiments from 2000 in Switzerland, 26 CFPs, including a phosphonate-based product, were evaluated, with the latter showing a significant reduction of PLB [15]. Salts of phosphonic acid, such as potassium phosphonate, are not of natural origin [16], and residues of potassium phosphonate were found in grapes, wine, potato tubers and other crops [17]. Hence, although these residues are considered to be of no toxicological relevance, phosphonate-based products were not recommended for organic agriculture [4].

In our own studies, the three botanicals, GC, RP and FA, showed the best potential [10] and were thus chosen for further evaluations under field conditions. Based on new and intensive discussions about the suitability of phosphonates for organic production in Germany 2010 [18], we commenced an approach to compare the efficacy of these promising botanicals and a potassium phosphonate-based product with that of copper. The main objective of this study was to investigate whether and how PLB could be effectively controlled in organic potato production without the utilisation of environmentally hazardous copper products.

## 2. Materials and Methods

### 2.1. Fungal Isolate and Inoculum Production for In Vitro Assays

For the in vitro assays, the *Phytophthora infestans* (Pi) isolate No. 01-001 from Agroscope Zurich-Reckenholz was used. The poly-spore strain was isolated from leaves of the Panda potato variety (low susceptibility to PLB) of a variety field trial at Reckenholz. The isolate was maintained on rye agar (200 g rye seed, 20 g agar agar, 5 g d-glucose, 1000 mL water) at 5 °C in the dark. Before the multiplication of the isolate for new experiments and to maintain its aggressiveness, it was employed after a host passage, utilising fresh potato slices [9].

### 2.2. Selection and Preparation of Antifungal Agents

The antifungal agents and the additive used for in vitro or field experiments are listed in Table 1. The products consisted of dried and cracked plant material of *Frangula alnus* (FA), *Rheum palmatum* (RP) and from *Galla chinensis* (GC). The product Phosfik^®^ (Ph, Biolchim Deutschland GmbH, Hannover, Germany) is registered in the EU as a foliar fertiliser in potatoes, applications of 2 to 3 L/ha are recommended in combination with Pi fungicides. Ph contains 440 g/L phosphonic acid (H_3_PO_3_) or 425 g/L of phosphite (PO_3_^3−^) [19]. It was suggested that the priming of defence reactions relies on these active compounds [20]. Potassium phosphonate can be a declared or undeclared component of foliar fertilisers or plant strengtheners that were authorised in organic farming in certain EU countries, but since 2014, its use is banned in organic farming in all EU countries [16].

In all experiments, the copper fungicides Kocide DF^®^ (KoDF, Bayer (Schweiz) AG, Münchenbuchsee, Switzerland) and Kocide Opti™ (Ko, Bayer (Schweiz) AG) served as positive controls. Both products are based on copper hydroxide and contain 40% (KoDF) or 30% (Ko) copper, respectively. The additive Nu-Film 17^®^ (NuF, Intrachem Bio (International) SA, Cologny, Switzerland) was added to the botanicals.

To prepare extracts and suspensions of the botanicals, the plant material (dried bark, roots and galls) was finely ground with a centrifugal mill in three steps to pieces of 2 mm, 0.5 mm and 0.25 mm (Retsch ZM 200, Schieritz & Hauenstein AG, Laufen, Switzerland). For the in vitro experiment, the plant powders were suspended and stirred at room temperature in sterile tap water with 10% ethanol over two hours. Subsequently, the extracts were filtered through a glass microfiber filter (Whatman^®^, 1820-060, Sigma-Aldrich, Buchs SG, Switzerland) and subsequently centrifuged for 10 min at 6000 rpm. For the sporangial germination experiment (see Section 2.3.1), the supernatant was additionally filtered through an Acrodisc^®^ syringe filter (Sigma-Aldrich Chemie GmbH, Buchs SG, Switzerland) with a nylon membrane (pore size of 0.2 µm; Sigma-Aldrich, Z259942, Buchs SG, Switzerland), and this last filtration was also carried out with the copper fungicide suspensions.

### 2.3. In Vitro Experiments

#### 2.3.1. Sporangial Germination

The effect of the antifungal agents on the germination of the sporangia by release of zoospores was assessed on microscope slides. On each slide, four silicon rings (Ø 1 cm) were placed and in each of them, 40 µL of the antifungal extracts or suspensions were added. To simulate the drying process on a leaf surface, slides were allowed to dry for 24 h and, subsequently, 40 µL of the sporangial suspension were added into the silicon rings. To induce the release of zoospores, the slides were incubated for 24 h at 4 °C. Thereafter, the emptied sporangia were counted with the aid of a microscope. Based on the germination rate of a water control, the relative percentage of germination was calculated [9]. Three independent experiments, each with four replicates per treatment and with sporangia densities of 1.0 × 10^5^, 1.25 × 10^5^ and 5.0 × 10^5^ sporangia/mL were conducted. For each experiment, freshly prepared extracts were used. The germination data (percentage of emptied sporangia) of the treatments were pooled and the means and standard errors were calculated.

#### 2.3.2. Mycelial Growth

The effect of the antifungal agents on mycelial growth was assessed with an agar diffusion test in Petri dishes (94 × 16 mm; Greiner Bio-One, St. Gallen, Switzerland). An agar plug of Ø 6 mm from a ten day old culture of Pi was placed with the mycelium side facing the agar in the middle of a Petri dish containing rye agar. In a distance of 15 mm from the central plug, six holes were cut with a cork borer (Ø 6 mm). In each of the six cavities, 70 µL of the extracts were added. The Petri dishes were subsequently incubated at 18 °C in the dark. After eight days, the radius of the mycelium was measured and placed in relation to a control treatment with water. The experiment was repeated three times with four rye agar dishes per treatment. The data of the three experiments were pooled and expressed as mean percentage of the radial growth in relation to the water control.

### 2.4. Field Experiments

From 2010 to 2013, field experiments at the Agroscope research stations Reckenholz near Zurich (47°25.8′ N, 8°31.2′ E; 443 m above sea level) and Tänikon near Ettenhausen (47°28.8′ N, 8°54.0′ E; 536 m above sea level) were conducted to investigate the effect of copper-free products (CFPs). Locations, varieties and treatments for the experimental years are listed in Table S1 and Table 2.

In all years, the effect of the antifungal agents was tested with the potato varieties Agria and Nicola (both intermediate susceptibility to PLB) and, in one case in 2012, with the variety Bintje (high susceptibility). The field experiments with six (2010–2012) or five (2013) treatments were conducted using a split-plot design with four blocks (replicates), with the “product treatment” as the main plot factor and two varieties as the subplot factor. In contrast, for the variety Bintje at the Reckenholz location in 2012, a randomised complete block design with four blocks was used. The plot size was 5 × 1.5 m (2 rows, one variety) or 5 × 3 m (2 × 2 potato rows, two varieties). The plots were flanked by the Panda variety with a low susceptibility to PLB. Two rows with Bintje between the experimental plots served as disease spreader rows. In the front and at the backside of the experimental plots, two rows with Panda were planted, whereas on the outer side, two rows with Bintje were planted. When the mean disease severity on the foliage in the spreader rows reached a level of 5 to 15%, these rows were mechanically defoliated. Row width was 75 cm and the distance between the 15 plants of a row was 33 cm.

For the field experiments, the same procedure to grind the plant material was applied as for the in vitro tests. The suspensions of the botanicals were prepared and stirred with tap water with 10% ethanol in the field using a battery driven drill machine. In 2010, an additional FA suspension treatment (FA+, Table 2) was prepared by stirring in water with 10% ethanol during two hours in the laboratory, before it was transported to the experimental site and this treatment was compared with a FA spray broth stirred solely at the field site.

The applications of the botanicals and the antifungal agents were carried out with a knapsack sprayer (Birchmeier, Stetten, Switzerland) with a spray-boom of 3 m with seven TeeJet (TeeJet Technologies GmbH, Ludwigsburg, Germany) 80° or six TeeJet 110° nozzles (extended range flat spray tips, TeeJet grey XR8006 or six blue XR11003, respectively): For the copper fungicides and Ph, the grey TeeJet nozzles with 1000 L/ha broth at 1.5 bar were used whereas for the preparations with botanicals or combinations of them with other products such as copper, the blue TeeJet nozzles with 690 L/ha broth at 3.0 bar were used.

For copper fungicides and Ph, only tap water was used. To avoid a blockage of the nozzles, all suspensions were filtered through cheesecloth before application. In all experiments, the effect of the antifungal agents was compared with an untreated control and a reference treatment with a copper fungicide. The first applications were conducted when the first PLB attacks were registered in Switzerland by the Swiss PhytoPRE PLB forecasting system [21]. The applications were performed once a week and, depending on the weather conditions, a total of eight to 10 applications were carried out.

The leaf blight disease severity was assessed by estimating the percentage of diseased leaf area for each plot as soon as the mean disease severity in the untreated plots reached 3–5%. Afterwards, the disease severity was rated every three to five days and for each plot, the area under the disease progress curve (AUDPC, [9]) was calculated. To estimate and compare the effect of the treatments, the relative AUDPCs based on the untreated control treatment (AUDPC = 1.0) were calculated for each variety.

To quantify phosphonate residues, tuber samples from untreated plots, from treatments with Ph, as well as from treatments with Ph and copper (only 2012), were taken from the field experiments of 2012 and 2013. Tubers were analysed at Interlabor Belp AG (Belp, Switzerland). The samples were homogenised and aqueous potassium phosphonate was extracted in water of 90 °C, filtered and purified by solid phase extraction. The phosphite ions (PO_3_^3−^) in the extract were measured with an ion-chromatography and conductivity detector.

### 2.5. Statistical Analysis

The data of the three in vitro experimental runs were pooled and mean values and standard errors of the treatment means are given. The AUDPCs and tuber yields of a given year were analysed with three-way (treatment, location and/or variety [if applicable] and replicates) ANOVAs. If the overall effect of a tested factor was significant in ANOVA, an all-pairwise multiple comparison procedure according to Tukey (α = 0.05) was conducted in order to evaluate differences between treatment means. To display the effect of the treatments on the two locations and the potato varieties, the mean values with the standard errors of the means are presented in the figures. To best illustrate the effect of the treatments on the AUDPC and on the potato tuber yield, all results are shown in relation to the corresponding control treatment. Linear regressions were calculated to display the relation between the amount of applied phosphite on the potato foliage and the respective content in the tubers. The statistical analyses were conducted using SigmaPlot^®^ Version 13 (Systat Software Inc., San Jose, CA, USA).

## 3. Results

### 3.1. In Vitro Experiments

**Figure 1:**
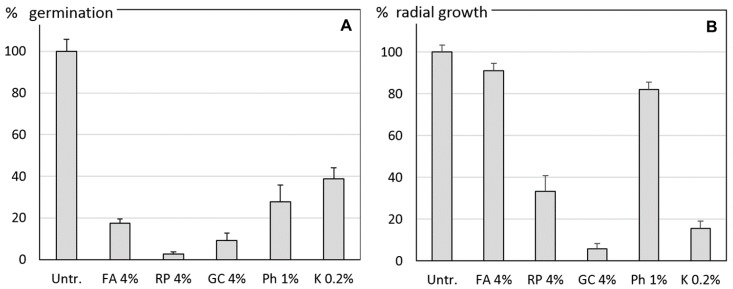
In vitro experiments on (**A**) the mean germination of *Phytophthora infestans* (Pi) sporangia and (**B**) the radial mycelium growth of Pi. Effect of 4% suspensions of the botanicals *Frangula alnus* (FA), *Rheum palmatum* (RP) and *Galla chinensis* (GC), of a 1% suspension of Phosfik^®^ (Ph) and of 0.2% Kocide DF (K). Untr. = Untreated control with water. Error bars indicate the standard errors of the means of three experimental runs with four replicates for each treatment.

All tested agents reduced the sporangia germination rate of Pi. The three botanicals reduced the mean germination rate by 82 up to 97%, Ph by 72% and KoDF by 61%. (Figure 1A). The mean germination rate of the Pi sporangia in the water control treatment was 57%. In the mycelium growth experiment (Figure 1B), the botanical FA and Ph reduced the radial growth of Pi only by 9% and 18%, respectively, whereas the copper fungicide and GC reduced it between 84 to 94%. With RP, a growth reduction of 67% was measured.

### 3.2. Field Experiments

#### 3.2.1. Field Experiment 2010

The onset of the late blight epidemic in 2010 in Switzerland was late. Moreover, a hot and dry period occurred between the second half of June until 22 July [22]. The field experiment at Reckenholz was not considered for this study since the PLB severity at the end of July was only 5% in the untreated controls of both cultivars, and no significant differences were observed for the AUDPCs and the tuber yields.

**Figure 2:**
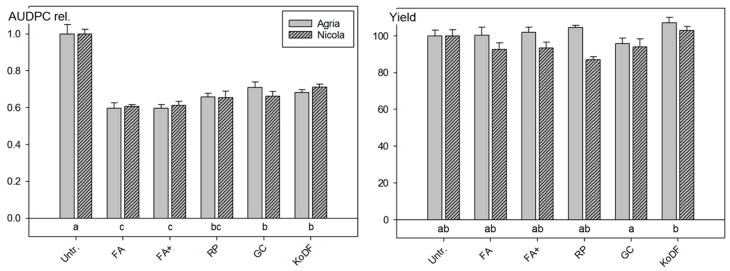
Field experiment in 2010 at Tänikon with the two potato varieties Agria and Nicola: Effect of suspensions of botanical powders of *Frangula alnus* (FA & FA+), *Rheum palmatum* (RP) and *Galla chinensis* (GC), as well as the copper fungicide Kocide DF (KoDF) on foliar blight (relative AUDPC) and the potato tuber yield (%). Treatments are described in Table 2. Untr. = Untreated control. Bars: Mean values of the varieties and standard errors of the means. AUDPC rel.: Area under the disease progress curve relative to the untreated control. Treatments labelled with the same letter are statistically not different (Tukey test *p* < 0.05).

In Tänikon, the disease onset was one month earlier and the first PLB lesions were found on 22 June (Table S1). The experiment focused on the three botanicals, FA, RP and GC, as well as the effect of an extensive preparation of FA with two hours stirring in the laboratory (FA+). All treatments reduced the AUDPCs significantly (*p* < 0.001) between 29% and 40% compared with the untreated control. The PLB reducing effect of the botanicals was equal to those of the copper treatment and the effect of the two FA treatments was even 10% higher (*p* < 0.05) (Figure 2). No difference was observed between the effect of FA and FA+. However, none of the treatments significantly increased the tuber yield compared with the untreated control (Figure 2 and Table S2).

Leaves of the variety Nicola, but not those of Agria, showed significant (*p* < 0.05) strong and medium phytotoxicity responses following RP and FA treatments, respectively [23]. In 2010, a negative, but not significant, effect on tuber yield of Nicola was observed following treatments with RP (13% reduction), FA (7%) and FA+ (7%) (Figure 2).

The field experiment of 2010 revealed significant reductions of the AUDPC for all four botanical treatments. An additional stirring of the suspensions is not needed as both FA and FA+ treatments had the same effect on AUDPC and tuber yield. The experiment also indicated that in contrast to Agria, RP and FA can negatively affect the tuber yield of Nicola.

#### 3.2.2. Field Experiments 2011

In 2010, the expert discussion on “Plant Protection in Organic Farming—Problems and Solutions”, organised by the Julius-Kühn Institute (Federal Research Centre for Cultivated Plants, Braunschweig, Germany), was fully dedicated to pros and cons of phosphonates in organic agriculture [18]. Due to the high interest of representatives from organic organisations, research institutions, industry and farmers, a phosphonate treatment was included in our field experiments of 2011 in order to compare it with the botanicals FA, RP and GC (Table S3).

In 2011, the onset of the late blight epidemic started again rather late at the end of May, but during July and August the weather conditions were very favourable for the disease development [22]. At the experimental site at Tänikon, 13 rainy days (max. measured daily amount of rainfall with 50 mm, and 10 days with more than 10 mm rainfall) were registered between mid-June and mid-July. On 4 July, the first PLB lesions were found in the untreated control. On 12 July, the disease severity reached 11% for Agria and 14% for Nicola in the untreated control and, on 18 July, the disease severity was 82% and 86%, respectively. On 22 July, this high disease level was also reached in the best performing treatments, hence, the last application planned for 25 July was omitted in Tänikon. At the experimental site of Reckenholz, the weather conditions were less conducive for PLB and the development of foliar blight was not as fast as in Tänikon.

**Figure 3:**
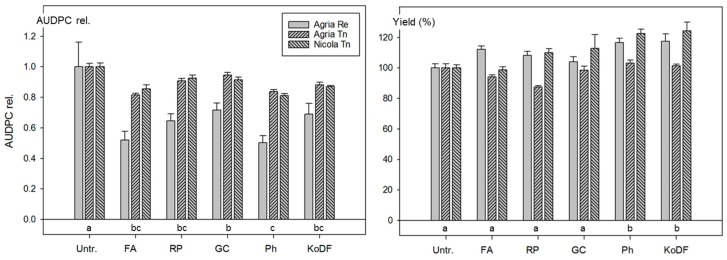
Field experiments in 2011 at Reckenholz (Re) with the potato variety Agria and at Tänikon (Tn) with Agria and Nicola: Effect of suspensions of botanical powders of *Frangula alnus* (FA), *Rheum palmatum* (RP) and *Galla chinensis* (GC), of a treatment with Phosfik^®^ (Ph) and of the copper fungicide Kocide DF (KoDF) on foliar blight (AUDPC). Treatments are described in Table S3. Untr. = Untreated control. Bars: Mean values of the varieties and standard errors of the means. AUDPC rel.: Area under the disease progress curve relative to the untreated control. Treatments labelled with the same letter are statistically not different (Tukey test *p* < 0.05).

The impact of the different weather conditions at the two experimental sites is reflected by the AUDPC data. At Reckenholz, the best performing treatments reduced the AUDPC of Agria by about 50%, whereas in Tänikon, the reduction for Agria and Nicola was below 20% (Figure 3). The effect on the AUDPC was significant (*p* < 0.05) for all treatments. The treatments with Ph, FA and RP performed as well as those with the copper fungicide (Figure 3 and Table S3). The impact of the treatments on the AUDPCs was similar for both varieties and both locations, however, this was not the case for the yield (Figure 3). The coefficient of correlation for the AUDPC and the yield data was *r* = 0.85 for the treatment averages of Agria at Reckenholz and *r* = 0.63 for those of Nicola in Tänikon, but only *r* = 0.04 for Agria in Tänikon. For data pooled over both locations, significantly (*p* < 0.05) higher yields resulted with KoDF (14%) and Ph (14%) compared with the untreated control (Figure 3 and Table S3).

The field experiment of 2011 showed that the efficacy of the treatments with Ph was as good as the one of the copper fungicide KoDF, with respect to the AUDPC and the yield. In addition, it revealed that the effect of the botanical FA on the AUDPC was as good as those of Ph or of KoDF, however, no increase in yield was detected.

#### 3.2.3. Field Experiments 2012

During the growing season 2012, weather conditions were highly favourable for late blight and a high number of attacks was recorded by the end of June [24]. In the region of our field experiments, the first PLB attacks were detected at the end of May.

At Reckenholz, the potato varieties Agria and Bintje and, at Tänikon, Agria and Nicola were planted. Residues of the phosphite ion (PO_3_^3−^) were analysed in tuber samples from all treatments with Ph, the untreated control and from the treatment with copper (KoDF). Treatments are described in the Table S4.

**Figure 4:**
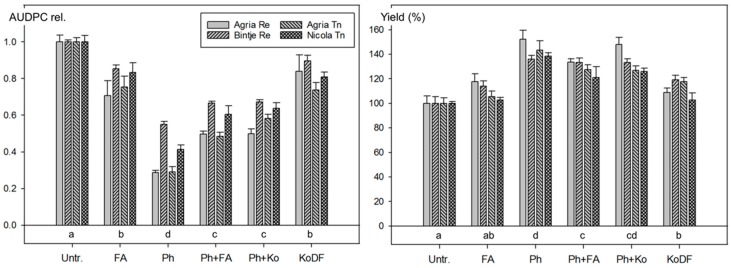
Field experiments in 2012 at Reckenholz (Re) with the potato varieties Agria and Bintje and at Tänikon (Tn) with Agria and Nicola: Effect of *Frangula alnus* (FA), Phosfik^®^ (Ph), Ph followed by FA (Ph + FA) or Kocide DF applications (Ph + Ko) and of Kocide DF (KoDF) on foliar blight (AUDPC) and on yield. Treatments are described in Table S4. Untr. = Untreated control. Bars: Mean values of the varieties and standard errors of the means. AUDPC rel.: Area under the disease progress curve relative to the untreated control. Treatments labelled with the same letter are statistically not different (Tukey test *p* < 0.05).

In contrast to the field experiment of 2011, strong effects of the treatments were not only observed on the reduction of foliar blight (AUDPC) but also on tuber yield. The reduction of the AUDPC was lower for the highly susceptible variety Bintje compared with that for Agria (Figure 4). As in the field experiments of 2010 and 2011, the impact of FA and KoDF on foliar blight was similar. However, the greatest reduction of AUDPC was observed with eight Ph applications in the Ph treatment (58%), followed by those applications with each four Ph, and then four FA (41%) or by four Ph and then four KoDF treatments (38%). Data on yields reflected well the AUDPC-reducing effect. Overall, the highest yield was registered with Ph, followed by the combined treatments Ph + FA and Ph + Ko. The phosphonate alone reduced the AUDPC by 58% and increased the yield by 43% compared with the untreated control and performed significantly (*p* < 0.001) better than KoDF (reduction of AUDPC and yield improvement by 17% and 12%, respectively) (Figure 4 and Table S4).

**Figure 5:**
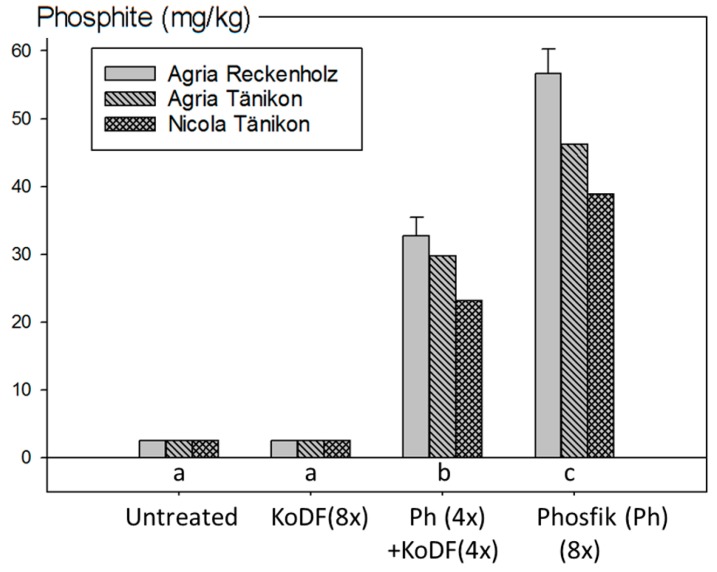
Residues of phosphite (PO_3_^3−^) in untreated and treated potato tubers from the field experiments in 2012. Tubers of the following treatments were analysed: Kocide DF (KoDF) alone (eight applications), Phosfik^®^ (Ph) (four applications of 3 L/ha) followed by KoDF (four applications) or Phosfik^®^ (Ph) alone (eight applications with 3 L/ha) (Table S4). Samples were taken at Reckenholz from Agria and at Tänikon from Agria and Nicola. Bars represent the mean values of the varieties and standard errors of the means. For “Untreated” and “KoDF(8×)”, concentrations were below the limit of detection (LOD = 5 mg/kg) and half of the LOD was plotted. Treatments labelled with the same letter are statistically not different (Tukey test *p* < 0.05).

With four and eight treatments of 3.0 L/ha Ph, mean phosphite concentrations in the potato tubers of 28.6 and 47.2 mg/kg, respectively, were measured. No phosphite was detected in untreated samples and those treated with KoDF and half of the limit of detection (LOD 5 mg/kg) is displayed in the figure. The results revealed a clear dependency to the amount of applied Ph, independent of the experimental location of sampling and the variety (Figure 5).

The results of the field experiment 2012 demonstrate that the effect of FA on foliar blight and on tuber yield was similar to those of the copper fungicide KoDF. Significant and far better results than with KoDF were achieved with eight applications of 3.0 L/ha Ph. The performance of four Ph applications followed by four FA or KoDF applications was lower than that with eight applications of Ph alone, but significantly higher than eight applications with KoDF. The content of phosphite residues in potato tubers was directly proportional to the amount of Ph applied on the potato foliage.

#### 3.2.4. Phosphite Residues from the Field Experiments in 2013

The aim of these experiments was to collect more information about phosphite anion PO_3_^3−^ residues from Ph treatments. Field experiments with the potato varieties Agria and Nicola were conducted at Reckenholz and Tänikon. To obtain unbiased residue data, a total of 2.700 g/ha copper was applied together with Ph in all treatments, except for the untreated control.

**Figure 6:**
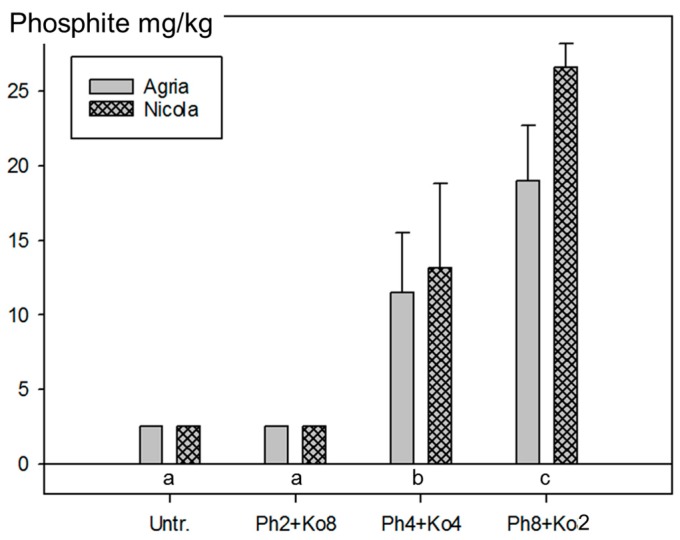
Residues of phosphite (PO_3_^3−^) in potato tubers from the field experiments in 2013 with two (Ph2 + Ko8), four (Ph4 + Ko4) and eight (Ph8 + Ko2) applications of 1.5 L/ha Phosfik^®^ (Ph) on the potato foliage and the untreated control (Untr.). Treatments are described in Table S5. Bars represent the mean values from samples of Agria and Nicola at Reckenholz and Tänikon (two samples per treatment) and standard errors of the means. The samples consisted of a mixture of tubers from the four replicates. Since samples from the untreated control (Untr.) and the treatment with two Ph applications (Ph2 + Ko8) were below the limit of detection (LOD = 5 mg/kg), half of the LOD was plotted. Treatments labelled with the same letter are statistically not different (Tukey test *p* < 0.05).

Treatments with two, four or eight applications of Phosfik^®^ were followed by eight, four or two applications with Kocide Opti™ (Ko; more recent formulation of copper hydroxide) were conducted, and only small, non-significant (*p* > 0.05), differences in AUDPC and yield effects were observed (Table S5). As in 2012, the content of PO_3_^3−^ in the tuber samples of the three treatments with Ph showed a clear correlation with the applied amount of the product. The application of four or eight times 1.5 L/ha Ph resulted in residues of 12.3 and 22.8 mg/kg PO_3_^3−^, respectively. The residues for the treatment with two Ph applications were below the limit of detection (LOD) of 5 mg/kg (Figure 6).

**Figure 7:**
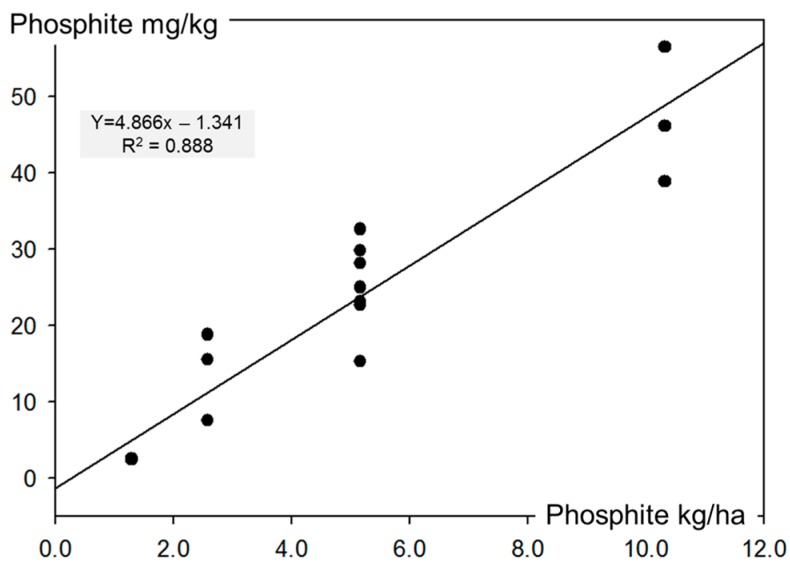
Correlation between the total amount of phosphite (PO_3_^3−^) applied with Phosfik^®^ (*x*-axis) and the measured phosphite residues in potato tubers (*y*-axis) from the field experiments in 2012 (*n* = 6) and 2013 (*n* = 12). Four samples with 1.29, seven with 5.16, four with 2.58 and three with 10.32 kg PO_3_^3−^/ha, respectively.

The results from 2013 correspond well to those of 2012. However, since, in 2013, four or eight times 1.5 L/ha Ph was applied and not 3.0 L/ha, as in 2012, the phosphite content in the potato tuber was also about half the contents of 2012. Significant differences were observed between the treatments (*p* < 0.05), but not for the experimental site and the varieties (*p* > 0.05). The strong correlation between the amount of phosphite applied on the potato foliage and the content in the tubers is shown in Figure 7, where the results of both years are given.

Overall, the results of the residue analyses revealed that the content of phosphite residues is directly linked to the amount of Phosfik^®^ applied on the potato foliage (*R*^2^ = 0.89 for pooled residue data from 2012 and 2013) and that this effect is independent of the timing of the applications and the potato variety.

## 4. Discussion

### 4.1. Disease Control with Botanicals

#### 4.1.1. Disease Control

In the current study, the potential of alternatives to replace copper fungicides for control of PLB in organic potato production was evaluated with in vitro tests and with multi-year field experiments.

The European Union proposed for 2002 a ban of copper-based fungicides as cited in Bassin and Forrer 2001 [15]. This suggested but not yet enacted ban initiated several research studies to identify copper alternatives. Through in vitro assays and partially with greenhouse experiments, promising substances were found, however, with the exception of products based on phosphonates, all of them failed under field conditions [7,8,9,15]. At Agroscope, preliminary field experiments showed encouraging results, when suspensions instead of ethanol extracts from the botanicals FA and RP were applied to control PLB [10]. In the field experiments from the current study of 2010 and 2011, significant reductions of PLB (AUDPC) were not only observed with FA and RP, but also with GC. In 2010 and 2011, these three botanicals were evaluated in subsequent experiments and compared with the effect of the copper fungicide KoDF. Compared with the untreated control, FA, RP, GC and KoDF reduced the AUDPC by 37%, 22%, 19% and 38%, respectively. Out of the tested botanicals, FA showed the best performance on PLB reduction and it was as efficient as KoDF, of which on average 2.2 kg of copper per hectare were applied.

The control of a fungal disease with the three tested botanicals in the current study is not restricted to PLB. On detached grapevine leaves, suspensions with 0.5% FA, RP and GC reduced the rate of *Plasmopara viticola* infection by 23%, 49%, 100%, respectively, and the sporulation on leaves was reduced by 90%, 100% and 95%, respectively [11]. Furthermore, in field experiments with Fusarium head blight (FHB) on wheat artificially inoculated with *Fusarium graminearum* and *F. crookwellense*, FA and GC reduced the content of the mycotoxins deoxynivalenol (DON) and nivalenol in the harvested wheat grains by 50 to 60% [25]. In fact, the performance of FA in mycotoxin reduction was statistically not different from that of a synthetic fungicide. Good results (reduction of 65% in DON content in grains of the FHB susceptible wheat variety Levis) were also achieved in a FHB field experiment with semi-natural inoculations. Besides the effect on FHB fungal species, seed coatings with GC proved to effectively control *Microdochium majus*, the pathogen causing snow mould disease in wheat, under field conditions [26].

#### 4.1.2. Effect on Yield

Though GC, RP and, especially, FA effectively controlled PLB, their impact on potato tuber yield was not satisfying. Whilst the yield in the field experiments from 2010 and 2011 was improved by 9.5% with the copper fungicide KoDF, applications of FA had no effect on the yield. However, in 2012, a year with an early onset and favourable conditions for PLB epidemics, FA increased the yield by 9% and was thus nearly as efficient as the KoDF treatment with 12%. The field experiment of 2010 at Tänikon revealed differences in the yield of the varieties since the effect of FA and RP was indifferent or positive for Agria but slightly negative for Nicola. Considering all experiments with the varieties Agria or Bintje, FA and KoDF improved the yield from 2010 to 2012 by 9% and 11%, respectively, compared with the untreated control. In three experiments with Nicola, the tuber yield with KoDF was 10% higher than in the untreated control, whereas FA even slightly decreased the mean yield (−2%). The reason for substantial disease control (AUDPC) but yield reduction for Nicola is not fully understood.

In the abovementioned studies to control FHB on wheat and grapevine downy mildew with FA, RP and GC, it was shown that the mode of action of FA and RP, but not that of GC, is based on induced resistance [11,25]. In the in vitro tests of the current study, GC almost completely inhibited the radial growth of Pi, RP reduced it by 70%, and FA only by 10%. Therefore, we assume that the mode of action of the three botanicals in PLB control is similar to that in grapevine or FHB control. The abovementioned decreasing yield effects following treatments with FA and RP could be due to the induction of defence mechanisms in the potato plants. In fact, it is known that active defence and its maintenance requires energy and nutrients [27], which in turn might lead to reduced vegetative growth and thus yield. Negative side effects of induced resistance were also observed with *Penicillium chrysogenum* treatments to control downy mildew in grapevine: The fungus not only induced resistance to the disease but also led to undesirable phytotoxicity [28]. In our study, negative effects on yield were clearly observed with the variety Nicola, but not or only slightly with Agria.

### 4.2. Disease Control with Phosfik^®^

From all products evaluated in the current PLB field experiments, Ph demonstrated the best performance. In 2011, the effect of eight Ph (1.5 L/ha) applications on foliage disease and on tuber yield was similar to those of eight KoDF applications (0.3 kg copper/ha). In 2012, with a severe PLB epidemic, the effect of eight Ph applications (3.0 L/ha) on foliage disease and on tuber yield was far better than those of the copper (8 × 0.3 kg/ha) treatments and those of the FA 4% suspensions. In line with these results, Borza et al. [29] observed a proportional relationship between the amount of applied phosphite based products and the resulting effect on PLB. In addition, 16 PLB field experiments in Peru, Ecuador, Kenya and Nepal, conducted by the International Centre of Potato CIP, demonstrated a clear relationship between the phosphonate application rate and the efficacy of PLB control. With eight applications of phosphonate salts at 2.5 kg/ha, as in the current study of 2012, their performance was similar to that of the conventional contact fungicides mancozeb and chlorothalonil used at comparable rates [30].

The good performance of phosphonates is based on various modes of action: Phosphonates are rapidly absorbed by plants, translocated into the phloem and the xylem, stimulate the defence responses of the hosts following infection and slow down the pathogens’ growth and inhibit its sporulation [31]. Furthermore, the reduction of the pathogens’ growth rate allows the host defence system additional time to stop the invading organism. In potato leaves treated with potassium phosphonate, a priming or rapid accumulation of hydrogen peroxide and superoxide anion production after Pi inoculation and earlier callose deposition was observed as compared with leaves treated with water [32].

#### Phosphite Residues in Potato Tubers

Residues of phosphite (PO_3_^3−^) were analysed in potato tubers from the field experiments in 2012 and 2013. As observed in another study [33], the phosphite concentrations in the tubers were directly proportional to the number of applications and the amount of applied Ph. With eight applications of 3.0 L/ha Ph, 47 mg/kg PO_3_^3−^ were detected in the analysed potato tubers. With four instead of eight applications, the concentrations were reduced by 40%. Indeed, a correlation coefficient of 0.94 between the total amount of phosphite applied and the residues in the tubers was obtained. In field trials in Sweden with up to twelve applications of Proalexin^TM^, a product based on potassium phosphonate, residues of 27 to 205 mg/kg PO_3_^3−^ were measured and the rates of phosphite levels in the tubers corresponded to 12 to 35% of the applied PO_3_^3−^ [34]. In our experiments, the range of these rates varied only between 15 to 18%.

Regardless of this undesirable property of phosphite based products, increasing attention to integrate them in PLB control strategies was observed in conventional agriculture, for example, in developing countries [30] as well as in Sweden [34] and in Canada [33]. It was underlined that due to their excellent environmental profile, the usage of such products will reduce the environmental impact of pesticides applied to the potato crop [35]. The European Food Safety Authority (EFSA) supports such positive appreciations: In the conclusions of the EFSA, low toxicity in mammalians was reported for potassium phosphonates. In addition, it is supposed that levels of potassium ions added to soil are within naturally occurring levels of potassium in mineral soils, and it was thus postulated that risks for fish and other aquatic organisms, honeybees and arthropods, earthworms and soil microorganisms, as well as on non-target terrestrial plants, are low [36]. Furthermore, the US Environmental Protection Agency (US-EPA) classified phosphonic acid (H_3_PO_3_) and mono- and di-potassium salts of phosphorous with low toxicity for the environment as biopesticides [37]. Nevertheless, the European Commission requested the EFSA to perform a dietary risk assessment of the maximum residue levels (MRLs) of various commercial formulations of phosphonates in a large number of crops. For potato tubers, the EFSA proposed a temporary MRL of 20 mg/kg for phosphonic acid [38]. Applying this MLR to the results of the field experiment in 2013, four but not eight applications of 1.5 L/ha Ph would not exceed this MLR level as the mean values for phosphorous acid detected in these treatments were 12.8 and 23.7 mg/kg, respectively.

Together with the favourable environmental profile, the complex mode of action of phosphonates is another reason to suggest the use of such products. Indeed, with the increasing occurrence of resistance to systemic fungicides based on phenylamides, the usage of phosphanates became more attractive [30,33]. Another interesting feature of foliar phosphonate applications consists in the suppression of tuber infections [37]. These features together with the possibility to reduce the amount of conventional fungicides and copper could contribute to develop PLB control strategies in integrated pest management (IPM) systems and indicate that potassium phosphonate could play a major role in control of both foliar and tuber blight [34]. Still, the toxicology and the environmental fate of formulated products should be investigated.

## 5. Conclusions

Our approach revealed for the first time the true potential of selected botanicals to replace or reduce copper in organic potato production under field conditions. In 2012, a year with a strong late blight epidemic, suspensions of finely ground bark of buckthorn (*Frangula alnus*, FA) controlled potato leaf blight (PLB) and led to tuber yields similar to those of eight applications with 0.3 kg/ha copper. In the field experiments in 2010 and 2011, the control of PLB with FA was equivalent to that of copper, however, the obtained yield data were not satisfying. Considering all three experimental years, we showed that the yield response to FA depends on the chosen potato variety: When yield data of the variety Nicola were excluded from the comparison with copper treatments, the performance of FA was equivalent to the one of copper. Hence, the replacement of copper is per se possible, but the suitability of various potato varieties has to be investigated in order to ensure stable yields. Since the effect of FA is most probably based on a priming or induction of self-defence mechanisms, studies to elucidate this suggested mechanism would be necessary. With deepened knowledge of the requirements and the dynamics of the defence process, optimised input strategies could be developed. The goal of organic production to produce and protect potatoes with natural and non-toxic products instead of copper seems to be realistic, but still affords further research.

With products based on phosphonates, equivalent and enhanced PLB control, as with copper or synthetic fungicides, is feasible. However, with respect to the MRL of 20 mg/kg of phosphorous acid in potato tubers, the number of applications would be restricted to two or three. The use of phosphonates could be beneficial for the environment and would also help to prevent fungicide resistance to Pi in conventional or IPM potato production, hence it is remarkable that so far their use in conventional and IPM late blight control plays a minor role. On the other hand, Phosfik^®^ and other products based on phosphorous acid or potassium phosphonate have been registered as fertilisers but not as fungicides, despite the fact that they do not contain plant nutrients [19,39]. A strict classification as fungicides would not only help to avoid their inappropriate use but could also stimulate further research upon their characteristics. Such knowledge could be employed best together with PLB forecasting systems such as PhytoPRE [21]. In the meantime and to reduce the risk of health and environmental problems due to synthetic fungicides and copper, further research is needed towards the development of botanicals, such as the promising bark of buckthorn.

## Figures and Tables

**Table 1:** Antifungal agents and additive evaluated for their effect against *Phytophthora infestans* in laboratory and field experiments.

Product Group/Agents	Abbreviation	Main Component	Provider
**Botanical (Name of Plant/Name of Drug)**			
*Frangula alnus*/Frangula cortex	FA	powder ^2^ of alder buckthorn bark	Hänseler AG (CH)
*Rheum palmatum*/Rhei radix	RP	powder of RP roots	Hänseler AG (CH)
*Galla chinensis* ^1^	GC	powder of GC galls	Berg Apotheke Zürich (CH)
**Phosphonate**			
Phosfik^®^ 3.27.18	Ph	3% N, 27% P_2_O_5_, 18% K_2_O ^3^	Biolchim Hannover (DE)
**Fungicides**			
Kocide DF^®^	KoDF	synthetic, 40% copper	Bayer (Schweiz) AG (CH)
Kocide Opti™	Ko	synthetic, 30% copper	Bayer (Schweiz) AG (CH)
**Additive**			
Nu-Film 17	NuF	resin of American pine	Intrachem Bio (Int.) SA (CH)

^1^ GC are galls of *Rhus chinensis* Mill. induced by aphid larvae. ^2^ Finely ground from dried and cracked plant material. ^3^ Including also microelements (all ≤ 0.02%).

**Table 2:** Overview of the standard treatments, the botanicals and the antifungal agents applied in the field experiments from 2010 to 2013 (number of applications is described in Table S1).

Year	2010	2011		2012		2013 *	
Location (Tänikon & Reckenholz)	Tän.	Tän.	Rec.	Tän.	Rec.	Tän.	Rec.
**Standards**							
Untreated	x	x	x	x	x	x	x
KoDF 200 g/ha Cu	x						
KoDF 300 g/ha Cu		x	x	x	x		
KoOpt (Ko) **						x	x
**Botanicals and phosphonate**							
*Frangula alnus* 4% (FA)	x	x	x	x	x		
*Frangula alnus* 4% (FA+)	x						
*Galla chinensis* 4% (GC)	x	x	x				
*Rheum palmatum* 4% (RP)	x	x	x				
Phosfik^®^ 1.5 L/ha (Ph)		x	x			x ***	x ***
Phosfik^®^ 3.0 L/ha (Ph)				x	x		
4× Ph 3.0 L/ha, then 4× FA (4%)				x	x		
4× Ph 3.0 L/ha, then 4× KoDF ****				x	x		

FA+: Suspension of FA stirred for 2 h in the laboratory before transportation to the field. KoDF: Kocide DF^®^; KoOpt: Kocide Opti™. * The aim of the experiment in 2013 was to obtain unbiased results with respect to PO_3_^3−^ residues. ** In all treatments except “untreated”, a total of 2700 g/ha Cu was applied. *** Treatments with two, four and eight applications of Ph. **** KoDF with 300 g/ha Cu per application.

## Supplementary Materials

The following are available online at www.mdpi.com/2309-608X/3/4/65/s1.
