# Supplementary material for: Botanicals and Phosphonate Show Potential to Replace Copper for Control of Potato Late Blight
Source: J Fungi (Basel). 2017 Nov 24;3(4):65. doi: 10.3390/jof3040065 (PMC5753167; doi:10.3390/jof3040065)
Supplement: Supplementary file 1 [file jof-03-00065-s001.pdf]

Supplementary Materials: Botanicals and Phosphonate Show Potential to Replace Copper for Control of Potato Late Blight

Table S1. Overview of locations, varieties and cropping factors from the potato field experiments 2010 to 2013.

Year	Location	Experiment		Varieties									
		Treatments	Replicates	Agria	Nicola	Bintje	Planting Date	Nb. Applications	First Application	Last Application	Date of Harvest	1st Lesion at Experimental Site *	1st Lesion in Treatment Plot
2010	Tänikon	6	4	x	x		23.04.10	10	4.06.10	4.08.10	2.09.10	22.06.10	25.06.10
2011	Tänikon	6	4	x	x		19.04.11	7 *	7.06.11	18.07.11	17.08.11	4.07.11	4.07.11
	Reckenholz	6	4	x			20.04.11	8	7.06.11	25.07.11	19.08.11	8.07.11	11.07.11
2012	Tänikon	6	4	x	x		30.04.12	8	7.06.12	25.07.12	17.08.12	7.06.12	15.06.12
	Reckenholz	6	4	x		x	3.05.12	8	5.06.12	17.07.12	15.08.12	6.06.12	15.06.12
2013	Tänikon	5	4	x	x		26.04.13	10	5.06.13	10.08.13	24.09.13	4.07.13	-**
	Reckenholz	5	4	x	x		26.04.13	10	5.06.13	10.08.13	11.09.13	28.06.13	-**

* Eight applications were planned in 2011. However, since in all treatments the disease severity of foliar blight was above 80% at the location Tänikon before the last application, this application was omitted. ** no information available.

Table S2. Effect of antifungal agents on foliar blight (AUDPC) and the yield of potatoes in a field experiment in 2010 at Tänikon. Data are mean values of the results with the potato varieties Agria and Nicola.

No.	Abbre-viation	Treatment/Agent	Number of Applications/Dosage	AUDPC (rel.)	T5%	Yield (t/ha)	%	T5%
1	Untr.	Untreated	none	1.000	a	41.8	100	ab
2	FA	*Frangula alnus*	10 × FA (4%)	0.601	c	40.4	97	ab
3	FA+	*Frangula alnus*	10 × FA+ (4%)	0.605	c	40.9	98	ab
4	RP	*Rheum palmatum*	10 × RP (4%)	0.655	bc	40.2	96	ab
5	GC	*Galla chinensis*	10 × GC (4%)	0.685	b	39.6	95	a
6	KoDF	Kocide DF	10 × 0.2 kg Cu ha^{-1}	0.697	b	43.9	105	b

The suspension of the treatment 3 (FA+) was stirred during 2 h in the laboratory before transportation to the field site. Values with the same letters are not statistically different (T5%: Tukey test $p < 0.05$).

Table S3. Effect of antifungal agents on foliar blight (AUDPC) and the yield of potatoes in a field experiment in 2011 at Reckenholz and at Tänikon. Data are mean values of the results with the potato varieties Agria at Reckenholz and Agria and Nicola in Tänikon.

No.	Abbre-viation	Treatment/Agent	Number of Applications/Dosage	AUDPC (rel)	T5%	Yield (t/ha)	%	T5%
1	Untr.	Untreated	none	1.000	a	33.9	100	a
2	FA	*Frangula alnus*	8 × FA (4%)	0.736	bc	34.5	102	a
3	RP	*Rheum palmatum*	8 × RP (4%)	0.834	bc	34.4	101	a
4	GC	*Galla chinensis*	8 × GC (4%)	0.867	b	35.5	105	a
5	Ph	Phosfik®	8 × 1.5 l Ph ha^{-1}	0.722	c	38.5	114	b
6	KoDF	Kocide DF	8 × 0.3 kg Cu ha^{-1}	0.821	bc	38.5	114	b

Values with the same letters are not statistically different (T5%: Tukey test $p < 0.05$).

Table S4. Effect of *Frangula alnus* and Phosfik® on foliar blight (AUDPC) and the yield of potatoes in field experiments in 2012 at Reckenholz and Tänikon. Data are mean values of the results with the potato varieties Agria and Bintje at Reckenholz and Agria and Nicola at Tänikon.

No.	Abbre-viation	Treatment/Agent	Number of Applications/Dosage	AUDPC (rel)	T5%	Yield (t/ha)	%	T5%
1	Untr.	Untreated	none	1.000	a	23.6	100	a
2	FA	*Frangula alnus*	8 × FA (4%)	0.798	b	25.8	109	ab
3	Ph	Phosfik®	8 × 3.0 l Ph ha^{-1}	0.420	d	33.7	143	d
4	Ph+FA	Phosfik®/*F. alnus*	4 × Ph, 4 × FA (4%)	0.589	c	30.3	128	c
5	Ph+Ko	Phosfik®/Kocide DF	4 × Ph, 4 × KoDF	0.623	c	31.3	133	cd
6	KoDF	Kocide DF	8 × 0.3 kg Cu ha^{-1}	0.829	b	26.4	112	b

In treatments 4 and 5, Phosfik® was applied first four times, followed by four applications with FA or KoDF. The dosages of Ph, FA and Ko were equal to those in treatments 2, 3 and 6. Values with the same letters are not statistically different (T5%: Tukey test $p < 0.05$).

Table S5. Effect of Phosfik® applications followed by applications with copper on foliar blight (AUDPC) and the yield of potatoes. Field experiments in 2013 at Reckenholz and Tänikon. Data are mean values of the results with the potato varieties Agria and Nicola.

Abbre-viation	Treatment/Agent	Number of Applications/Dosage	AUDPC (rel)	T5%	Yield (t/ha)	%	T5%
Untr.	Untreated	none	1.000	a	35.3	100	a
Ph2+Ko8	Phosfik®/ Koc.Opti	2 × 1.5 l Ph ha^{-1}+ 8 × 338 g Cu ha^{-1}	0.428	bc	40.9	116	b
Ph4+Ko6	Phosfik®/ Koc.Opti	4 × 1.5 l Ph ha^{-1} + 6 × 450 g Cu ha^{-1}	0.474	bc	38.6	109	ab
Ph8+Ko2	Phosfik®/ Koc.Opti	8 × 1.5 l Ph ha^{-1} + 2 × 1350 g Cu ha^{-1}	0.557	b	40.7	115	b
Ko	Kocide Opti	10 × 270 g Cu ha^{-1}	0.390	c	40.0	113	b

In all treatments except "untreated", 2700 g Cu/ha was applied in total with Kocide Opti™ (Koc.Opti). Values with the same letters are not statistically different (T5%: Tukey test $p < 0.05$).
